# Identity without milestones: a narrative inquiry into the rites of passage shaping professional identity formation in health professions educators

**DOI:** 10.1080/10872981.2026.2696154

**Published:** 2026-07-08

**Authors:** Farah Ennab, Mersiha Kovacevic, Shaista Salman Guraya

**Affiliations:** a Institute of Learning, Mohammed Bin Rashid University of Medicine and Health Sciences, Dubai Health, Dubai, United Arab Emirates

**Keywords:** Professional identity formation, educator identity, health professions education, medical educator, narrative inquiry

## Abstract

**Introduction:**

Professional Identity Formation (PIF) in medicine is typically supported by certain milestones and well-researched rites of passage. In contrast, educators who enter Health Professions Education (HPE) often do so without such signposts, inhabiting a liminal space where identity is constantly negotiated. Despite the growing availability of HPE academic programs, little is known about how these programs shape educators’ identities or which experiences serve as significant identity-forming moments. Our study aims to identify the transformative ‘aha’ moments, which can serve as rites of passage, helping to strengthen the health professions educators’ identity.

**Methods:**

Guided by a social constructionist stance, we employed narrative inquiry to explore the stories of 16 healthcare professionals enrolled in, or recently graduated from HPE academic programs. Semi-structured interviews were conducted and analysed using a re-storying technique. Transformative Learning Theory (TLT) informed the understanding of cognitive, emotive, and affective dimensions of identity shifts, which were captured through the lens of epiphanies.

**Results:**

Through the analytical interpretation of these stories, we identified four epiphanies. The ‘power of people’ in the form of competent faculty to develop educational alliances, which influenced all dimensions of TLT. The ‘Language of HPE’, articulated through paradigmatic orientations and vocabulary, instilled cognitive shifts. The ‘burden of tasks’, reflecting the distinct nature of the HPE-related assignments, exerted emotional weight. Finally, the ‘sense of belonging’ through live social interactions between faculty and peers, strengthened the modes of belonging.

**Conclusion:**

Identification of these transformative ‘aha’ moments provides clear, measurable steps into how educator identity formation can be fostered and strengthened in the context of HPE academic programs. Such insights call for thoughtful faculty selection, improved curriculum structuring, and scheduling practices. By honoring the educators’ stories, we give voice to their lived realities and recognize the significance of HPE academic programs as beautiful avenues for identity formation.

## Introduction

Health Professions Education (HPE) remains a field shaped by uncertainty, diverse entry pathways, and evolving professional boundaries. It is often perceived that individuals stumble into this field, drawn by disillusionment with clinical work, professional burnout, or a search for intellectual fulfilment beyond patient care [1–3]. However, pathways into HPE are diverse, with some individuals entering through longstanding interests in teaching, educational leadership, academic scholarship, or non-clinical educational roles. Health professions educators are sometimes portrayed as occupying liminal spaces between clinical, educational, and institutional roles within healthcare systems and academic cultures [4,5]. Their identities are often viewed as secondary or appended to primary clinical or disciplinary roles, despite their important contributions to healthcare education [6].

Professional Identity Formation (PIF) refers to the adaptive process through which individuals integrate and internalise the norms, values, and beliefs of a profession [7]. In medicine, clinicians in training acquire their professional identity through structured transformative stages, gradually learning to think, feel, and act like physicians [8–10]. It initially begins with their roles as students. Then, as they transition to interns, the boundaries between learning and practice blur, and the *self* begins to merge with the role, deepening the different dimensions of what it means to ‘become’ a practicing clinician. However, the PIF of health professions educators appears less structured and considerably more ambiguous. Thus, the formation and development of educator identities cannot be left to happenstance. Educational programmes oriented toward pedagogical practices in HPE may therefore play an important role in nurturing this emerging identity [11]. Such programmes commonly include postgraduate certificates, diplomas, and master’s degrees designed to support healthcare professionals (HCPs) in developing expertise in teaching, assessment, curriculum, leadership, and educational scholarship. These qualification-oriented HPE programmes have become increasingly widespread across academic health systems and universities internationally [12].

Identity formation is punctuated by certain critical incidents, commonly referred to as ‘rites of passage’ [13]. Defined as ‘*rites which accompany every change of place, state, social position, and age*’ [14]. These experiences can be seen as critical moments of transition. In most instances, when individuals move from one identity to another, these are the places of liminality, or the *‘betwixt and between’* phase [15]. These moments challenge educators’ assumptions about themselves and their roles, creating periods of vulnerability during which identity is most open to transformation. Such periods of vulnerability are marked by cognitive dissonance, emotional toll, and reconciliation between changing realities [9]. The nature and significance of those critical incidents differ considerably across various rites of passage, depending on the type of learner, training stage, contextual setting, and the precipitating cause. The identity formation experienced by undergraduate medical students [13] and clinicians [16] has been widely researched through this notion. Typically, the rites of passage that scholars know and celebrate are situated within clinical programmes, such as medical school curricula or clinical residency training. These include many events like the white coat ceremony, a surgical resident's first appendectomy, witnessing a natural birth for the first time, or the initial encounters with cadaveric dissections in anatomy labs. However, when we speak of educator identity, these rites of passage or identity-forming and deforming incidents remain largely unexplored, despite the recognised value of HPE academic programmes. We know that such programmes create spaces to nurture the educator identity of HCPs and support them to develop educational scholarship, build pedagogical expertise, establish professional networks, and gain confidence and credibility [11,17]. All of which are essential elements to support educator identity formation within academic and clinical educational settings.

But how can we study these rites of passage among health professions educators? In this study, we focus on HCPs enroled in postgraduate HPE academic programmes who were navigating evolving educator roles across clinical, academic, and allied health settings. Many of these experiences unfolded within multicultural HPE environments, particularly in the United Arab Emirates (UAE), where educators frequently negotiate diverse professional, cultural, and institutional expectations. These contextual dynamics may shape how transformative moments and educator identities are experienced and interpreted. Transformative Learning Theory (TLT) provides a useful theoretical framework through which we can see the cognitive, emotive, and affective components occurring within these rites of passage [18]. Through this perspective, moments of uncertainty, dissonance, and realisation may be understood as transformative ‘aha’ moments that reshape how individuals think, feel, and act as educators [19,20]. The applicability of TLT has extended across different academic disciplines, including HPE [21]. Hence, TLT offers a valuable framework for exploring how HCPs critically reassess, reshape, and transform their educator identity. This transformation, however, is neither linear nor effortless. The journey of adult learners in HPE academic programmes is influenced by moments of newness, uncertainty, and challenges [22]. Despite the increasing availability of HPE academic programmes, little is known about the transformative moments that shape educator identity formation within these spaces. In this study, we therefore sought to identify the transformative ‘aha’ moments, that may function as rites of passage in the development of educator identity. To address this, we posed the following questions:

1. How do HPE academic programmes shape the professional identity of health professions educators?
2. What transformative moments within HPE academic programmes shape educator identity formation?

## Methodology

This empirical study adopted an exploratory qualitative design and was rooted in a social constructionism paradigm, which embraces the belief that ‘*multiple realities exist, realities that are dependent on interactions between the individual and the social world; the individual and the social world are deeply entangled through dialogue*’ [23]. By positioning our work within such an orientation, we examined how individuals construct their realities of educator identity in a situated context. Recognising our own role as researchers who actively co-construct these realities alongside participants, through the analytical interpretation of their stories.

### Methodological approach

Narrative inquiry [24], an established qualitative approach that has been increasingly used within HPE research [25–27], guided this study to explore how health professions educators narrated the contextual, relational, and temporal dimensions of educator identity formation. Using this approach, stories were viewed as a means through which individuals make sense of, and communicate their lived experiences [28,29]. Narrative inquiry attends closely to ‘three commonplaces’: temporality (time), sociality (personal and social conditions), and place (contexts in which stories unfold) [30,31].

### Participants recruitment

A criterion-based sampling strategy [32] was used for recruitment, requiring participants to meet predefined inclusion criteria relevant to the research question. Eligible participants were HCPs who were either: a) actively enroled in a postgraduate HPE academic programme, or b) had graduated from such a programme within the previous six months. Participants were also required to have prior teaching, educational, or learner-related experiences that enabled reflection on their evolving educator identity. Eligibility was initially confirmed through self-report during recruitment and subsequently clarified through follow-up communications prior to interview scheduling.

Participants in our study were from diverse professional backgrounds and held clinical, academic, and administrative positions across various institutions, with differing levels of involvement in teaching and learning practices. Most participants were enroled in UAE-based programmes, with a few from the UK, experiencing diverse learning formats ranging from hybrid (face-to-face and asynchronous online components) to purely online programmes. While recruitment was initially facilitated through institutional networks, snowball sampling [33] enabled access to participants across multiple HPE programmes and institutional settings, thereby capturing a broader range of educational experiences, professional roles, and learning environments. An overview of participants’ professional characteristics is provided in Table 1.

**Table 1.** Summary of participants’ demographic and professional characteristics.

Pseudonym*	Gender	Age Range	Professional Role	Teaching/learning Experience*	Programme Format*	Country
Sara	F	<30	Non-clinical medical educator	E	Hybrid	UAE
Layla	F	40+	Administrator	E	Hybrid	UAE
Adam	M	40+	Dentist	M	Hybrid	UAE
Sami	M	40+	Surgeon	M	Hybrid	UAE
Maha	F	40+	Endocrinologist	M	Online	UK
Sophie	F	40+	Family medicine physician	S	Online	UK
Farida	F	40+	Nurse	E	Hybrid	UAE
Yazan	M	30–39	Simulation educator	M	Hybrid	UAE
Sharon	F	30–39	Respiratory therapist	E	Hybrid	UAE
Khaled	M	30–39	Emergency physician	M	Hybrid	UAE
Nada	F	30–39	Family medicine physician	E	Hybrid	UAE
Amir	M	30–39	Non-clinical medical educator	M	Hybrid	UAE
Hala	F	<30	Resident physician	E	Online	UK
Khadijah	F	40+	Family medicine physician	S	Hybrid	UAE
Zahra	F	40+	Internal medicine physician	S	Hybrid	UAE
Karim	M	30–39	Non-clinical medical educator	M	Hybrid	UAE

Notes: *Pseudonyms were used to protect participants’ identities and ensure anonymity. *Hybrid format refers to programmes that include both face-to-face and online components (with synchronous and asynchronous learning activities), whereas online format refers to programmes delivered entirely online. *Teaching and learning experience is categorised as E (early career, 0–4 years), M (mid career, 5–9 years), and S (senior, ≥10 years), reflecting participants’ cumulative involvement in formal educational roles within clinical or academic settings. UAE: United Arab Emirates, UK: United Kingdom.

The first author (FE) distributed invitations to participate in semi-structured interviews via official institutional emails to students enroled in HPE academic programmes at their institution, and they subsequently shared the invitation with their colleagues enroled in similar programmes at other institutions. This approach supported the recruitment of participants with varied professional and educational experiences. The recruitment process took place over approximately two weeks, and no incentives were offered. All participants provided written informed consent prior to the commencement of their interviews, including written consent for anonymised narrative excerpts to be included in research dissemination and publication. This observational study was conducted in accordance with the ethical standards outlined in the Declaration of Helsinki [34].

### Ethical approval

Ethical approval was granted by the Mohammed Bin Rashid University Institutional Review Board (Reference #MBRU IRB-2025-280) and by the Dubai Scientific Research Ethics Committee, Dubai Health Authority (DSREC-SR-04/2025_05).

### Data collection

Data collection took place between May and July 2025. Semi-structured interviews [35] were conducted by FE, who was concurrently enroled in an HPE programme and therefore occupied an insider-outsider position within the educational context. Interviews were conducted in a dialogic and reflective manner, encouraging participants to narrate critical experiences and transformative moments associated with evolving educator identity [36]. Probing questions were used iteratively to clarify meanings, assumptions, and emotional responses emerging during the narratives. Given the reflective nature of narrative inquiry, attention was paid to participant comfort, emotional sensitivity, and voluntary disclosure during interviews. Prior to data collection, a reflexive mock interview was conducted with the senior author (SSG) to refine question flow, interviewing approach, and management of potentially sensitive discussions. This interview was conducted solely for preparatory purposes and was not included in the analysis. All interviews were conducted online, each lasting between 45 and 60 minutes, and were audio-recorded and transcribed using the Microsoft Teams platform. Throughout the study, participants were reminded that they could decline to answer questions or withdraw at any stage.

All interviews were conducted in English. English was the language of instruction across the HPE academic programmes represented in this study and constituted a core admission requirement for participants. All participants had demonstrated English language proficiency through institutional admission processes and were working in multicultural healthcare environments where English served as the primary language of professional communication. Consequently, interviews were conducted and transcribed directly in English, and no translation procedures were required.

The focus of our study was to capture the particular ‘aha’ moments or rites of passage in educators’ academic journeys; therefore, we carefully devised an interview guide, which was informed by the principles of TLT (see Supplementary File). The questions were aimed at eliciting narrative accounts, resting on the premise that individuals, when adequately and carefully prompted, are inclined to share their personal stories [37]. Interviews explored participants’ educational backgrounds, experiences within HPE programmes, and critical moments that shaped changes in how they understood and enacted their educator roles.

### Analytical approach

Analysis in narrative inquiry is anchored in the storyteller’s perspective, recognising that the stories themselves constitute the data [38]. During analysis, episodes described as moments of realisation, dissonance, self-questioning, and shifts in perspective were examined iteratively through TLT constructs to understand how participants cognitively, emotionally, and behaviourally negotiated changes in their educator identity. However, in our analysis, we observed a fluid connection between *‘*epiphanies’, as described by Denzin [39], and the various elements of TLT to make sense of the data. Epiphanic moments served as key analytic entry points through which transformative shifts were identified and interpreted. In this way, TLT functioned as an interpretive and conceptual lens guiding the coding and meaning-making process, rather than merely being applied retrospectively to emergent themes. Epiphanies closely align with TLT, as both highlight moments of critical reflection, prompting significant shifts in participants’ perspectives and evolving professional identities. Such moments represent what we may also describe as transformative ‘aha’ moments [20]. According to Denzin, epiphanies are aptly described as ‘*interactional moments and experiences which leave marks on people’s lives. In them, personal character is manifested. They are often moments of crisis. They alter the fundamental meaning structures in a person’s life’* [40].

Remaining close to the data, we used verbatim excerpts from participants' words and personal experiences. Furthermore, to ensure participants’ anonymity, pseudonyms have been used throughout all narrative accounts. A re-storying technique [41] commonly employed in narrative inquiry analysis, was followed to analyse the stories, consisting of three detailed steps (See Table 2). In this ‘re-storying’ process, the researchers take an active role in interpreting the participants’ stories to draw profound insights and answers [42].

**Table 2.** Detailed analytical steps undertaken using the re-storying technique [41].

Re-storying process	Description
**Step 1:** Familiarisation with data	Each transcript was carefully read multiple times to fully grasp the various experiences of all participants coming from different backgrounds and contexts, ensuring that no voice was overshadowed. Our initial impressions and observations were noted separately after reading, documenting all details, which were relevant to research question and objectives.
**Step 2:** Identifying story turning points	The key turning points (‘aha’ moments) identified as ‘epiphanies’ were inductively coded within each story, using a colour-coding approach to organise and compare emerging patterns across stories. Coded excerpts were then organised and grouped according to the specific type of epiphanies identified in the stories, where each epiphany amplified a particular dimension of TLT. Patterns across grouped epiphanies were analysed, allowing us to understand how HPE academic programmes could shape the professional identity of health professions educators.
**Step 3:** Writing	In the writing and analysis process, we ensured to remain close to the participants’ original words, feelings, and viewpoints. Direct verbatim excerpts were presented with each story to support the analytical patterns identified in each section. Key ‘aha’ moments were re-storied, describing the power of the identified epiphanies, which could be viewed as transformative experiences or rites of passage, helping to strengthen their educators’ identity.

Following discussions with the research team, we reached a consensus to read the data with this classification in mind. This layered approach, intertwining epiphanies and TLT (see Table 3), is essential, as it guided our interpretation of the stories.

**Table 3.** Classification of Epiphany types and their alignment with TLT dimensions.

Epiphany type	Definition and relationship to TLT elements
1. Cumulative—Power of people	A profound change that occurs within one’s life. It arises from the accumulation of significant interrelated experiences, influencing individuals profoundly across emotive, cognitive, and affective dimensions.
2. Illuminative—Language of HPE	Particular incidents or events in our lives that reveal sudden insights or realisations, triggering moments of cognitive shifts.
3. Major—Burden of tasks	Detrimental and deeply unsettling moments, shaking one's confidence, and carrying an emotional intensity.
4. Relived—Sense of belonging	Moments that individuals often seek to revisit, sometimes repeatedly, in an attempt to make sense of them. These experiences tackle the affective dimension.

Note: Definitions of epiphanies are adapted from Savin-Baden and Niekerk [43].

### Trustworthiness and rigour

Using the widely established criteria identified by Lincoln and Guba [44], credibility, dependability, confirmability, and transferability were ensured. For credibility, we carefully revisited and re-read each participant's transcript multiple times to capture the nuances of their personal experiences that might otherwise go unnoticed. To further strengthen the dependability of our analysis, we detailed each step of the re-storying technique clearly and recorded an audit trail of our decisions. Confirmability was established through maintaining reflective notes throughout the analysis process and consciously checking our personal biases and assumptions to avoid altering or overshadowing any of the stories presented. Finally, for transferability, we aimed to create context-rich, thick descriptions for each story so that readers could resonate with the findings and determine some relevance within their own contexts. This rigorous and transparent approach [45] supported the trustworthiness of our work.

### Reflexivity

Interpretations in our study were influenced by the researchers’ proximity to the educational context. This proximity was most pronounced for the first author (FE), who was enroled in the MScHPE cohort from which most participants were recruited. Co-inhabiting this space provided familiarity with the programme’s norms, tensions, and expectations, thereby supporting close engagement with participants’ stories. At the same time, this required careful attention to how shared experiences could influence analytical decisions. Reflexivity was addressed through iterative dialogue within the research team. The senior author (SSG), who holds a faculty role within the programme, provided analytic rigour and consistently challenged emerging interpretations. The second author (MK), drawing on expertise in organisational learning research, contributed an additional perspective that supported interrogation of assumptions. Throughout the analysis, we adopted a social constructionist stance, recognising our active role in this process. Reflexive memoing, regular team discussions, and close attention to participants’ words were used to ensure that interpretations remained grounded in participants’ accounts rather than the researchers’ own experiences.

## Results

Using a narrative inquiry approach, we explored the stories of 16 HCPs enroled in HPE academic programmes. Across these narratives, participants described transformative ‘aha’ moments that altered how they understood themselves as educators, how they related to educational communities, and how they enacted educator roles within their professional contexts. Analysis of these stories revealed four interconnected forms of epiphanic experiences, interpreted through the lens of TLT: cumulative, illuminative, major, and relived epiphanies. Together, these epiphanies provided a framework for understanding how educator identity was validated, challenged, expanded, and reinforced through cognitive, emotional, and behavioural shifts experienced during participation in HPE academic programmes.

I.
**Power of people**

Participants described how interactions with faculty and peers profoundly influenced the emotive, cognitive, and affective dimensions of their experiences, as reflected across their narratives. The *‘power of people’* was an important relational influence in participants’ educator identity formation. In particular, the *‘characteristics of the teaching faculty’* and *‘socialisation with near peers’* were identified as key influences on participants’ identity development. Participants described how feedback, intellectual challenge, collaborative reflection, and peer discussions shaped their confidence, thinking, and evolving sense of themselves as educators. This was a profound hallmark of the hybrid HPE academic programmes.

i. *****Characteristics of the teaching faculty*****

Dr. Sara, an early-career medical educator enroled in a hybrid programme, described feedback from faculty as an important moment in developing confidence in her scholarly and educator identity. Receiving detailed feedback from experienced educators appeared to validate her thinking and helped her reconsider her role beyond task completion. Her narrative suggests that receiving feedback from experienced educators provided a sense of validation and reassurance, helping her develop greater confidence in her emerging educator identity. The accessibility and supportiveness of faculty also appeared to foster trust within the learning relationship. Dr. Sara recounts her academic journey, describing it as:

*‘You know, again the stages of what I went through to write the assignments took the brainstorming, the thinking, the constructing, the skeleton of what I want to add the references, and then the actual write up. So for me I do. I write it. I think it's a good job done, but that's my perception only. And then I submit, once I get the feedback for my assignment and that educator, in my opinion, full-fledged educator shares that. Thank You.”*

Dr. Layla, an administrator enroled in a hybrid HPE programme, further described how faculty members fostered growth by encouraging learners to navigate uncertainty independently rather than providing direct answers. Faculty who demonstrated trust in learners’ abilities, while simultaneously offering encouragement and reassurance, appeared to create a safe space for learners to think differently, challenge themselves, and develop greater confidence in their evolving educator identities. Although this process initially generated discomfort, gradually finding her own path forward became an important transformative experience in her educational journey. In her story, Dr. Layla shares:

*“But you will learn in the process like the way they designed you will learn during the whole process… facilitating things where you are not clear with all the things, but you are actually, you know striving to find and see … what is the best option for me … what are the possibilities … it gave me the confidence to think differently … try myself a bit harder and give me the challenges, the opportunities to learn and go back to the theory … So that's the beauty of the programme.”*

ii. *****Socialisation with near peers*****

Beyond the exchange of ideas, interactions with near peers appeared to shape how participants understood themselves as educators. Exposure to colleagues from diverse professional backgrounds challenged previously taken-for-granted assumptions about teaching and learning, encouraging participants to move beyond profession-specific identities and towards a broader educator identity. Through these interactions, participants began to see themselves as legitimate members of a wider educational community, reinforcing their sense of belonging to the field of HPE. In Dr. Adam’s story, who is a dentist with an active role in teaching dental trainees in clinical practice, the value of peer interactions, especially from diverse professional backgrounds, was palpable.

*“So we come somehow from different background… So that brought up different context to the discussions that triggered more interesting interactions. Between us as a learner.. it was completely it's a different approach compared to how we get within a programme, especially in undergrad level …. But when we moved on to the health professions education programme, it was a different story. You would feel that we were all in the same level, somehow just different roles. And that felt different. And also I felt like, OK, now it feels like we are all on the same boat trying to learn”*

Implicitly or explicitly, the social interactions we have with professionals who possess unique clinical and educational experiences nudge us to view our teaching philosophies through a different lens. Such candid exchanges among colleagues can become an essential source of support, fostering a sense of solidarity and shared purpose. This reflective dialogue presents informal learning opportunities and highlights how peer socialisation in the context of busy academic programmes and demanding clinical responsibilities can sustain motivation and positively shape the identities of health professions educators. This concept is expressed in Dr. Sami’s story, who is a practicing surgeon and is involved in teaching and learning practices of undergraduate medical students and residency programmes. He highlights the profound impact of near peer feedback loops in these academic programmes.

*“Moments from peer feedback that we used to have... I was sometimes sharing some, some opinions or some things with some of my peers and they were sharing with me and we were giving each other feedback specifically among each others... especially those who were involved in education more help me to understand and reinforce the values of collective learning and continuous growth in the programme and in medical education in general... One person I really believe have had a big impact on me, although I think this person did not feel that, but I learned a lot from him or her”*

II.
**Language of HPE**

The *‘language of HPE’*, reflected through illuminative epiphany, was identified within participants’ stories, triggering sudden moments of cognitive shifts. Two distinct triggers led to this realisation. First was the ‘*acquisition of language’*, and second was the ‘*practice of that language’*. This was a shared recollection among learners across all programmes, seen through their efforts to acquire, articulate, and practice the unique language of HPE during their enrolment. As HCPs accustomed to the scientific, quantitative, and clinical language of medicine, encountering new terminologies and glossaries represented critical turning points for these learners.

i. *****Acquiring the language*****

For many participants, acquiring the language of HPE represented more than learning new terminology. It functioned as an identity-forming process through which learners gained access to the discourse, assumptions, and ways of thinking that characterise the educator community. As participants became familiar with educational theories, paradigms, and conceptual vocabularies, they began to reframe their understanding of teaching from an intuitive activity to a scholarly practice, supporting their transition from clinician to educator. Dr. Maha, an endocrinologist who is enroled in a purely online programme with extensive clinical teaching experience of medical students and residents, distinctly remembers this moment. Referring to it as an expansive departure from the structured discourse of clinical medicine language.

*“It was during that time that I learned how expensive the theories in education were. And how there were different schools of thought? … OK. So at that moment it was and it was new to me because I as a physician, most of what I studied in medical school was science based. It was not literature. But to me. This, it seemed, reading through the the text it was not science as I knew it… And to me, this was an eye opener for me … It's a totally different world”*

ii. *****Practicing that language*****

Several participants described initially feeling overwhelmed by this language, yet gradually developing greater confidence and familiarity through continued engagement with educational literature and discussion. Unlike clinical protocols, which provide structured and clear directives, educational models often demand intensive cognitive and interpretive efforts. The openness about initially feeling overwhelmed captures an honest reality for many learners. Academic language can sometimes feel impenetrable, especially when compared to the everyday vocabulary of clinical medicine. For many HCPs grappling with this complexity, this practice demands patience, persistence, and even a bit of courage. Dr. Sophie, a senior family medicine physician enroled in a fully online programme, described the cognitive dissonance she experienced when first encountering educational theories and models that differed substantially from the scientific language of medicine.

*“You know, there was stuff like, you know, the [Kotter] eight step process for change or the current step process for this or that. And we really kind of heavily went it the reading for leadership was awful. It was really, really like opaque, completely different language to what we use in medicine and some of the papers. Oh my God, I'm not sure I understand this..… but we got there in the end”*

III.
**Burden of tasks**

The *‘burden of tasks’* was not experienced merely as academic workload. Rather, these moments exposed tensions between participants’ established professional identities and the expectations of becoming an educator-scholar. This was evident through the *‘nature of tasks’* and the *‘execution of such tasks’.* Academic writing, critical synthesis, and evidence-informed argumentation challenged previously secure perceptions of competence, creating periods of uncertainty during which participants questioned, renegotiated, and reconstructed aspects of their emerging educator identities.

i. *****Nature of tasks*****

Farida, a nurse, enroled in a hybrid programme, and is no longer actively involved in clinical teaching. She reflected on her early days in her HPE programme, particularly highlighting the struggle of being introduced to evidence-based writing styles and still learning how to train that academic voice. Her recollection of academic assignments was marked by a sense of surprise and overwhelm at the expected requirements and burden of tasks in a postgraduate programme. Her story reveals insights into how the pace and frequency of programme-related tasks may negatively impact the overall learning experience for some students. Such emotional turbulence may diminish learners’ enthusiasm moving forward, leaving them hesitant or reluctant to fully engage in future tasks.

*“We didn't tap into the concepts yet and it was very diverse and it was very quick and it was at the beginning. So maybe those were the challenging parts of it .. I did not expect to be that way .. It's worth mentioning that we were having assignments maybe every week, so every week or every, let's say, every week or every two weeks, you have to write several thousand, you know, thousand word or 2000 words. Along with reading a lot about the new topic to keep. And that was challenging and not easy”*

ii. *****Execution of tasks*****

Evidence-based practice requires an ability to maintain a neutral voice and to accurately cite the literature. However, learners often struggle to maintain this neutrality of academic voice. For Dr. Sophie, the necessity of scientific citations and the requirement for critical synthesis reveal a loud tension between her clinical identity and the academic identity she’s expected to carry and perform. The reality is that some clinicians, especially those who have long stepped away from formal academic writing, often find themselves feeling significantly disadvantaged, despite their clinical maturity. Tasks once familiar become daunting. Stirring anxieties and undermining their confidence and abilities to perform, for many learners, it’s in this very unsettling space that their educator identity is brought into question. In Dr. Sophie’s story, whose persona remains consistent from previous narration, she expresses frustrations of a slightly different nature. Her struggle was rooted in the execution of academic tasks. This discomfort resides not in this learner’s clinical expertise, but the disruption of previously stable professional competence.

*“You know, papers from management journals, which I found to be incredibly long. And I wasn't sure you know what to take from. What were the key points I was supposed to take? … I really hate, you know citations and referencing. I'm really not very good at all of that Stuff. .. You know where it came from or what the hell style of citation I'm supposed to use. And then the big challenge is synthesising that. But then that's a whole point that that's really to make us think critically. And justify why we agree or disagree. So that was challenging.”*

IV.
**Sense of belonging**

The *‘sense of belonging’* emerged as a relived epiphany within learners’ stories across the hybrid HPE programmes, deeply engaging the affective dimension of TLT. This sense of belonging was inculcated through the ‘*value of different perspectives’* and ‘*feeling of being heard in discussions’,* which fostered reflexivity and relational self-awareness. Within these spaces, learners felt their unique voices and diverse perspectives were heard, valued, and included in broader conversations. Each desire to deliberately return to these experiences enabled them to reflect on their evolving identities as educators, deepening their belonging to these communities of practice.

i. *****Value of different perspectives*****

The hybrid programmes take learners beyond merely fitting in. Such avenues provide reassurance to learners, affirming that there can indeed be many different ways of ‘*knowing and doing’*. Through opportunities to engage in real-time discussions, participants become psychologically reassured, feel valued, and develop the confidence to re-evaluate their educational philosophies. Rather than becoming trapped in loops of uncertainty and introspection, learners begin to embrace the richness that comes from contrasting perspectives, enriching their understanding and nurturing their evolving professional identity as educators. Dr. Yazan, enroled in a hybrid programme and actively engaged in simulation-based education, reflected on how discussions within the programme encouraged him to reconsider his assumptions about learners and interactions within educational settings. Engaging closely with others and participating in ongoing discussions sets the stage for internal dialogues about their interactions with learners in their own contexts. He highlights how such learning environments allow diverse perspectives to be valued, openly compared, and thoughtfully contrasted with those of others.

*“Well, it made me more mindful of different. Different opinions, different types of learners, different perceived attitudes. You know what attitudes I perceive from them? You know, made me think a little bit deeper when it comes to, you know, is this really a so and so attitude or am I just perceiving it as such? Are they perceiving? Is this because of something I did or said? So it it just made things a little more intentional for me as well... So I would say the impact of it is it's made me a little more honest even in the sense that I can. I can say, hey, listen, you know, is this what's happening or is there something else?”*

ii. *****Feeling heard in discussions*****

Sharon, a respiratory therapist enroled in a hybrid HPE programme, described how participation in group discussions gradually shifted her sense of belonging within educational spaces. Initially, speaking during discussions felt intimidating and unfamiliar. However, as her perspectives were acknowledged and included within conversations, she began to feel more confident, engaged, and connected to the learning community. Her narrative reflected how feeling heard within these discussions contributed to her evolving educator identity. It was this profound sense of ‘*feeling included”* that motivated Sharon and learners like her to continually seek out and revisit these moments.

*“I assumed always as I mentioned that the students come and teacher usually speaks, so that was a challenge for me to like speak up in the class… You hardly listen for 20% and the remaining is all like going up above your head, but then what? … So initially it was a challenge for me to focus completely in the class, but as discussion grew more interesting and I as I was responding and I was participating in those discussions and wanting it again, I felt myself included and I was like, there was no way to zone out”.*

## Discussion

Our study brings into focus those critical ‘aha’ moments that serve as rites of passage in educator identity formation for HCPs enroled in HPE academic programmes. Through participants’ stories, we observed how educator identity formation unfolded across cognitive, emotive, and affective dimensions, as clinicians, administrators, and academics questioned previously held assumptions, renegotiated established professional identities, and gradually came to see themselves as educators. These transformative shifts were often triggered by moments of dissonance, validation, belonging, and intellectual discovery that challenged familiar ways of ‘knowing and doing’. While illuminative and major epiphanies transcended across both hybrid and online programmes, cumulative and relived epiphanies were more commonly associated with hybrid learning environments, suggesting that socially situated and relational experiences may play an important role in educator identity formation.

### The educator behind the feedback

Faculty characteristics, particularly their ability to provide effective feedback, was perceived as an important influence on participants’ educator journeys. Feedback interactions often served as moments of validation and reassurance, especially as learners navigated unfamiliar educational expectations, academic writing practices, and evolving educator roles. Participants’ stories suggested that approachable, available, and supportive faculty contributed to the development of trust within the learning relationship, reinforcing learners’ confidence in their emerging educator identities. These findings resonate with the literature, emphasising the relational and psychologically safe dimensions of effective feedback skills in HPE contexts [46,47]. Within the socially intricate contexts of HPE academic programmes, delivering effective feedback requires more than subject expertise alone. While credibility, articulation, and contextual expertise are often valued qualities in educators, participants’ stories suggest that feedback provision also demands an understanding of psychological safety, learner agency, and relational trust. Urging us to treat it as a precious conversation, rather than a sheer commodity [48]. Clinical expertise alone cannot translate automatically into proficiency in providing actionable and effective academic feedback [49]. Clinicians, despite their interdisciplinary expertise, are seldom trained in the art of delivering effective feedback within educational contexts, especially with respect to its structure, content, and format [50].

Participants in our study were mature adult learners who already possessed deeply established professional identities grounded in medicine, allied healthcare, administration, and educational practice [11]. Re-entering academic spaces that demanded unfamiliar forms of scholarly writing, reflection, and knowledge synthesis appeared to place many learners within liminal ‘betwixt and between’ spaces of new communities of practice [15]. Within these transitions, feedback became more than a technical educational task. It functions as part of an ‘educational alliance’ [51]. shaping how learners interpreted their place within evolving educator communities. Published literature [51,52] highlights how learners actively assess their relationships with educators when deciding how to internalise, question, or resist feedback. This aligns closely with the principles of psychological safety and learner agency—the essential dimensions of feedback quality [53], making each feedback interaction a potentially identity-shaping moment, influencing how participants come to see themselves as educators and valued members of their educational communities [46].

### The language and paradigms of HPE

Another important finding was the role of HPE language and paradigmatic thinking as a rite of passage within educator identity formation. As per Krishnan’s framework [54], the presence of *‘specific terminologies and specialised theories and concepts’* constitutes one of the defining characteristics of discipline. Participants in our study described how entering HPE required engagement with unfamiliar educational terminology, theoretical frameworks, and scholarly traditions grounded in psycho-social sciences rather than biomedical discourse [55]. This transition often represented an illuminative epiphany, prompting participants to reconsider how knowledge, teaching, and educational practice could be understood. The interdisciplinary nature of HPE, described as ‘multidisciplinary edge effect’ [56], appeared to intensify these experiences. Participants encountered perspectives drawn from sociology, psychology, philosophy, and educational theory, which differed substantially from the positivist and post-positivist orientations commonly emphasised within clinical training [57,58]. For many learners, this shift generated cognitive dissonance as they attempted to move beyond binary understandings of knowledge toward more contextual, interpretive, and socially situated ways of thinking. The acquisition of HPE language, therefore, extended beyond learning new terminology; it involved entering a different discourse community and negotiating new ways of seeing, interpreting, and articulating educational experiences. Participants’ stories also highlighted the emotional and intellectual burden associated with this transition [59]. Academic writing, theoretical synthesis, and engagement with educational literature often functioned as a troublesome yet transformative ‘threshold concept’ [60], unsettling previously stable professional identities while simultaneously opening new possibilities for educator development. These threshold crossings carried both cognitive and affective demands, particularly for mature clinicians and allied HCPs returning to formal academic study after prolonged engagement in practice settings [61,62].

In their discussion, Brown et al [63]. draw on Varpio’s metaphor of the ‘glass box’, offering a useful way of understanding these tensions. Clinicians entering HPE programmes are not only introduced to new educational content; they are also asked to view familiar problems through unfamiliar paradigmatic lenses [64,65]. Participants in our study described struggling with shifts from generalisability toward contextualisation, from quantification toward qualitative interpretation, and from seeking definitive answers toward engaging with complexity, reflexivity, and multiple perspectives. These approaches are often unfamiliar within traditional clinical training, contributing to moments of uncertainty and epistemological tension, turning into a major epiphany that could feel overwhelming or even detrimental for the learners. Our findings therefore resonate with Mukhalalati, Elshami [66] who note that HPE programmes frequently struggle to integrate socially derived theoretical perspectives in ways that learners can meaningfully navigate and internalise.

### Strengthening the communities of practice

Hybrid HPE programmes were the avenues through which socialisation into new communities of practice occurred at a transformative pace. Wenger describes communities of practice as groups of individuals who share a concern or passion and learn to do it better through ongoing interaction [67]. In our view, simply inhabiting a space within a community of practice does not automatically lead to experiencing a deep sense of belonging nor the formation of an educator identity. Building on Wenger [67] seminal work, McMains, Durning [68], McMains, Konopasky [69] have described the three distinct modes of belonging - engagement, imagination, and alignment - which were strongly reflected within participants’ narratives. Together, these modes appeared to support movement from peripheral participation toward fuller participation within educator communities. When participants discover those modes of belonging, such experiences can be seen as another rite of passage. In our study, *‘engagement’* was evident through dialogues and social interactions, particularly when diverse perspectives were shared, heard, and valued. While *‘imagination’* appeared when learners recognised that educational problems could be tackled in many ways. They appreciated how each paradigm offered its own unique angle and how diverse worldviews can address the same issue differently. This recognition allowed participants to imagine themselves more confidently in educator roles. Finally, *‘alignment’* occurred when participants discussed and recognised the numerous possibilities available within HPE practices. Viewed through this lens, the prominence of cumulative and relived epiphanies within hybrid programmes highlights the importance of socialisation, dialogue, and live peer interaction in supporting educator identity formation.

### Implications for educational practice

Several practical implications arise from our study. Firstly, because faculty interactions emerged as important identity-validating experiences, we ought to think carefully about the type of faculty we recruit for HPE academic programmes. The teaching faculty should be confident, communicative, and well-versed in providing effective feedback to learners. Equally important, faculty should be cognisant of how educator identity transitions unfold in such settings, as these transitions can be complex, messy, and deeply personal. This calls for establishing clear criteria with specific standards to guide the faculty recruitment process. It may be that not everyone fits naturally into the role of an HPE educator. Therefore, we must look at their backgrounds, consider their research acumen, ability to create psychologically safe environments, and feedback literacy skills. Another critical implication relates to how programme content supports educator identity transitions. The content and programme-related tasks in such programmes should be informative, high-quality, and holistically designed, drawing insights from psychology, philosophy, and sociology disciplines. When we recognise and honour those disciplinary foundations, learners may better understand the complex realities of HPE.

Additionally, paradigms hold significant importance, as they constitute the fundamental building blocks of our practice [70]. If we do not reveal and teach the various paradigms in HPE, constructive alignment, both in educational interventions and research initiatives, is simply unattainable. Hence, educators and programme designers need to be intentional in clarifying and aligning those paradigms from the outset. Finally, we must rethink how we can build communities of practice that can be intentionally cultivated to support educator identity formation. Most of the programmes currently operate in online delivery formats, with such degrees often treated as ancillary or ‘add-on’ qualifications to HCPs [12]. However, we should reclaim and advocate for physical spaces, where rich discussions and social exchanges occur. Most online HPE programmes are asynchronous, which makes building a social community, a difficult task. These findings do not suggest the superiority of one delivery mode over another but rather, draw attention to the importance of intentional learning design [71]. In this context, purely online HPE programmes may benefit from incorporating design thinking principles that foreground interaction and live discussions, thereby broadening different perspectives. Finally, we also cannot safely assume that membership in academic groups will successfully form communities of practice. It is time that we take ownership of our disciplinary identity.

### Strengths and limitations

To the best of our knowledge, this study is the first of its kind to explore the PIF of health professions educators by combining narrative inquiry with TLT principles, situated within the context of HPE academic programmes. Previous research work has mainly relied on psychological or sociological frameworks, without adopting narrative inquiry as a methodological approach or explicitly anchoring their studies in TLT elements. Narrative inquiry was a powerful tool to tap into those critical moments, as it placed individual voices and deeply personal stories of identity shifts at the heart of our research process. Capturing lived experiences with an authenticity and depth that other methodological approaches may otherwise overlook. Moreover, our study fills a significant epistemological gap, as very few of the existing studies stem from our particular region of the world.

As with all narrative inquiry, the findings presented here were shaped through interpretive engagement between participants’ stories and the researchers’ analytical lenses. The researchers’ proximity to the educational context, particularly the first author’s insider familiarity with the MScHPE environment, supported a nuanced understanding of participants’ narratives and contributed to the verisimilitude of the findings. Although all participants were fluent English speakers and routinely used English within their academic and professional contexts, English was not the first language for many participants. It is therefore possible that certain culturally situated meanings, linguistic nuances, or emotionally nuanced expressions may not have been articulated with the same richness as they might have been in participants’ native languages. However, because participants were reflecting on experiences that occurred within English-medium HPE programmes and English-speaking professional environments, the potential impact of language on the narratives was likely mitigated.

At the same time, the epiphanies we identified stem from specific moments in time, whereas stories are evolving living structures. A longitudinal study might therefore be valuable, allowing exploration of how these epiphanies evolve, transform, or even shift over an extended period of time. Finally, while age, gender, and professional backgrounds are recognised as important dimensions of PIF, our analysis did not reveal discernible differences in how epiphanies were experienced across these characteristics. Instead, the identified epiphanies appeared to serve as shared rites of passage across all participants. This does not suggest that such dimensions are unimportant, but rather reflects a limitation of the current study design.

## Conclusions

HPE academic programmes can serve as profound avenues for educator identity formation. Within these spaces, educators experience transformative moments of visible tension, characterised as identity-forming incidents. In our narrative inquiry study, participants’ stories revealed that faculty teaching in the HPE academic programmes exert a strong influence. This is especially true through the effective feedback they provide. Additionally, acquiring the specialised language and glossary specific to HPE can be a daunting task, introducing tensions to this new emerging identity. Yet, within these challenging spaces lie opportunities for belonging. It was seen that encouraging diverse perspectives and creating opportunities for rich discussions can significantly strengthen educators’ sense of belonging to those communities of practice. Our research work offers health professions educators and programme directors practical insights distilled from the stories of those residing in it. These insights highlight the importance of recruiting faculty who can foster supportive and autonomy-enhancing learning relationships, designing curricula that intentionally scaffold learners through unfamiliar educational discourses and identity transitions, and pacing academic tasks in ways that acknowledge the emotional and cognitive demands experienced by working HCPs.

## Supplementary Material

Supplementary Material Interview_Guide_Supplementary_File.pdf

## Data Availability

The original contributions presented in the study are included in the article material; further inquiries can be directed to the corresponding author.
