# Supplementary material for: Identity without milestones: a narrative inquiry into the rites of passage shaping professional identity formation in health professions educators
Source: Med Educ Online. 2026 Jul 8;31(1):2696154. doi: 10.1080/10872981.2026.2696154 (PMC13353455; doi:10.1080/10872981.2026.2696154)
Supplement: Supplementary Material — Interview_Guide_Supplementary_File.pdf [file ZMEO_A_2696154_SM8030.pdf]

Supplementary File

Semi-structured interview guide

-In today's interview, I want to listen to your story of when you felt like an educator in this program, and to explore this further, I will be asking you a few questions. Shall we begin?

The participants will be asked a primary question: "**Tell me the story of when you started to feel like an educator during this program. Did it happen yet"?**

**Specific prompting questions will then follow:*

1. Can you provide us with some information about your HPE academic program? And what were your motivational factors for enrolling in this program?
2. What events or experiences in the HPE program have had the most impact on your attitudes about being a medical educator? Was there any specific learning activity that stood out?
3. Have you felt a significant challenge, struggle, or discomfort during the program that led you to critically reflect on your beliefs or assumptions about being an educator? How did you work through this, and how did it impact your development?
4. Have you experienced any key "aha" moments (instances of sudden clarity or realization) that significantly shaped your understanding of your role as an educator? What triggered these moments, and how did they influence your perspective?
5. Do you think people's perceptions of you as an educator have changed throughout your participation in the HPE program? How has that influenced your self-perception?
